# Physiological Profile of Neuropeptide Y-Expressing Neurons in Bed Nucleus of Stria Terminalis in Mice: State of High Excitability

**DOI:** 10.3389/fncel.2018.00393

**Published:** 2018-11-05

**Authors:** Achim Leonhard Walter, Julia Constance Bartsch, Maia Datunashvili, Peter Blaesse, Maren Denise Lange, Hans-Christian Pape

**Affiliations:** Institute of Physiology I, Westfälische Wilhelms-Universität Münster, Münster, Germany

**Keywords:** NPY, extended amygdala, stress disorders, BNST, anxiety, classification, patch-clamp electrophysiology, mouse

## Abstract

Both, the anterior bed nucleus of the stria terminalis (BNST) and the neuropeptide Y (NPY) system are involved in shaping fear and defensive responses that adapt the organism to potentially life-threatening conditions. NPY is expressed in the BNST but NPY-expressing neurons in this critical hub in the stress response network have not been addressed before. Therefore, we performed whole-cell patch-clamp recordings in acute slices of anterior BNST from *Npy*-hrGFP transgenic mice to identify and characterize NPY-expressing neurons. We show that NPY-positive and NPY-negative neurons in anterior BNST match the previous classification scheme of type I (Regular Spiking), type II (Low-Threshold Bursting), and type III (fast Inward Rectifying) cells, although the proportion of these physiological phenotypes was similar within both neuronal subpopulations. However, NPY-positive and NPY-negative neurons possessed distinct intrinsic electrophysiological properties. NPY-positive neurons displayed higher input resistance and lower membrane capacitance, corresponding to small cell bodies and shorter less ramified dendrites, as compared to their NPY-negative counterparts. Furthermore, NPY-positive neurons generated higher frequent series of action potentials upon membrane depolarization and displayed significantly lower GABA_A_ receptor-mediated synaptic responsiveness during evoked, spontaneous, and elementary synaptic activity. Taken together, these properties indicate an overall state of high excitability in NPY-positive neurons in anterior BNST. In view of the role of the anterior BNST in anxiety- and stress-related behaviors, these findings suggest a scenario where NPY-positive neurons are preferentially active and responsive to afferent inputs, thereby contributing to adaptation of the organism to stressful environmental encounters.

## Introduction

Neuropeptide Y (NPY) is a 36 amino acid neuropeptide, which exists across all vertebrates (Tatemoto, 1982). Together with PYY and PP, NPY belongs to one family of neuropeptides expressed along the gut–brain axis (Holzer et al., 2012). Its high conservation during evolution, and its involvement in pathophysiological mechanisms related to neuropsychiatric disorders, turns NPY into an important target in neurophysiological research (Heilig, 2004). A bulk of literature indicates an antidepressant- and anxiolytic-like effect of NPY (Heilig, 2004; Wu et al., 2011; Bowers et al., 2012). For instance, NPY is implicated in mechanisms of fear learning and memory (Heilig, 2004; Tasan et al., 2016). A central role of NPY in fear extinction is supported by the complete absence of fear extinction in mice lacking NPY (Verma et al., 2012). More recently it has been shown that various NPY receptor subtypes specifically contribute to fear memory and extinction (for review see: Tasan et al., 2016). Five different receptor subtypes, Y1, Y2, Y4, Y5, and y6, have been identified, with the latter not being functional in primates and humans (Michel et al., 1998). The Y1 receptors are typically located at postsynaptic sites and their stimulation in the basolateral complex of the amygdala (BLA) reduces anxiety-like behavior (Karlsson et al., 2008), most likely through membrane hyperpolarization of BLA principal neurons (Sosulina et al., 2008; Giesbrecht et al., 2010). The Y2 receptors are mostly of presynaptic nature regulating the release of GABA or glutamate (Silva et al., 2001; Ledri et al., 2011). Stimulation of Y2 receptors increases (Bacchi et al., 2006), whilst deletion of Y2 receptors reduces anxiety-like responses (Tasan et al., 2010). Fear memory and extinction, by comparison, are controlled by combined action of Y1 and Y2 receptors, as deduced from studies in mice lacking NPY and Y1/Y2 receptors (Verma et al., 2012).

Neuropeptide Y is generally expressed in dispersed interneurons, which are mostly of GABAergic nature and which often co-express SST (McDonald, 1989; Karagiannis et al., 2009; Tasan et al., 2016). In addition, NPY-immunoreactivity is found in long fiber tracts, such as the corpus callosum and stria terminalis, implying NPY expression also in projection neurons (Allen et al., 1983; Tasan et al., 2010). The influence of NPY on fear memory and extinction has been mostly related to sites of action in amygdalar circuits, such as BLA and central nuclei (CeA), although the exact cellular source of NPY remains to be delineated (Tasan et al., 2016). Furthermore, NPY is expressed in the BNST, with NPY-immunopositive staining existing along the stria terminalis and within neurons in the BNST (Allen et al., 1983; Pleil et al., 2012; Tasan et al., 2016). The BNST is part of the extended amygdala and considered a center for integration of signals carrying information about negative valence or anxiety-like states, and thereby being highly relevant for stress-related psychiatric diseases (Walker et al., 2009; Lebow and Chen, 2016).

The BNST can be roughly divided into anterior/posterior and dorsal/ventral sections through fiber bundles of the stria terminalis and AC, respectively (for review see Gungor and Paré, 2016; Lebow and Chen, 2016). In more detail, the BNST-AL and the BNST-AM are delineated laterally by the internal capsule and caudate putamen of the striatum, ventrally by the AC, dorsomedially by the septum and divided by fibers of the stria terminalis. The ventral part of the anterior BNST (BNST-AV) is seen as extension of BNST-AL and -AM ventrally to the AC. In BNST-AL, the majority of cells is GABAergic, whereas in BNST-AV both glutamatergic- and GABAergic cells seem to exist in roughly equal numbers (Nguyen et al., 2016). Electrophysiological studies suggested that different types of neurons exist in these BNST regions, which can be distinguished based on intrinsic membrane properties and response patterns in a behavioral context (Hammack et al., 2007; Rodríguez-Sierra et al., 2013; Daldrup et al., 2016; Daniel et al., 2017). However, the physiological properties of NPY-expressing neurons remain unknown.

In view of the critical role of both the BNST and the NPY system in anxiety-like and stress-related behaviors and associated disease states, we identified NPY-expressing neurons in BNST and compared their properties with those that lack NPY expression. We focused on the anterior part of BNST, given previous electrophysiological classification of neurons in this area and its role in the domain of fear (Lebow and Chen, 2016; Nguyen et al., 2016). We made use of a bacteria artificial chromosome (BAC)-*Npy* mouse with reliable expression of the GFP in NPY-expressing cells (van den Pol et al., 2009), thereby allowing the identification of NPY-positive neurons in slice preparations *in vitro*.

## Materials and Methods

### Animals

All electrophysiological procedures were performed in male (*n* = 44) and female (*n* = 44) 9- to 32-week old *Npy*-hrGFP transgenic mice (NPY-GFP mice). NPY-GFP mice were created by van den Pol et al. (2009), and the specificity of hrGFP-expression in NPY-neurons has been validated. The NPY-GFP transgenic mouse line, which is based on a C57BL6/J background, was obtained from Jackson Laboratory (JAX stock #006417; Bar Harbor, ME, United States). Animals were bred in-house, kept on a 12h-light-dark cycle and had access to food and water *ad libitum.* No more than five and no less than two mice were kept in a cage. All experiments were carried out in accordance with the European Community’s Council Directive of 22 September 2010 (2010/63/EU). Ethical approval for all mice used in this study was obtained from the Landesamt für Natur, Umwelt und Verbraucherschutz Nordrhein-Westfalen (LANUV NRW, Germany). The protocol was approved by the local authorities.

### Preparation of Acute Brain Slices

For preparation of brain slices, mice were anesthetized with isoflurane (1-chloro-2,2,2-trifluoroethyl difluoromethyl ether; 2.5% in O_2_; Abbott, Wiesbaden, Germany) and complete loss of reflexes was established, before decapitation. The skull was placed on a frozen metal plate (−18°C) and brains were removed rapidly. Brains were then put into ice-cold (0–4°C), oxygenated preparation solution containing (in mM): 2.5 KCl, 1.25 NaH_2_PO_4_, 10 MgSO_4_, 0.5 CaCl_2_, 20 piperazine-N,N′-bis(2-ethanesulfonic acid) (PIPES), 10 glucose, and 200 sucrose (pH 7.35; ∼300 mOsmol/kg). Coronal sections (300 μm), containing the BNST, were cut with a vibratome (Leica VT1200s, Leica, Wetzlar, Germany) at low speeds (∼0.05 mm/s). Slices were additionally divided along the dorsoventral axis, to separate the two hemispheres. Subsequently, slices were put into a pre-heated (∼34°C), gas-flushed (95% O_2_ and 5% CO_2_), incubation chamber, filled with preincubation solution containing (in mM): 125 NaCl, 2.5 KCl, 1.25 NaH_2_PO_4_, 24 NaHCO_3_, 2 MgSO_4_, 2 CaCl_2_, and 10 glucose (pH 7.35; ∼300 mOsmol/kg). Before electrophysiological recordings commenced, slices were allowed to equilibrate at room temperature for at least 1 h.

### Electrophysiological Recordings

Slices were placed in a recording chamber, which was continuously perfused (peristaltic perfusion pump, Watson-Marlow 205s, Watson Marlow GmbH, Rommerskirchen, Germany, ∼1.5 ml/min) with heated (∼32°C), oxygenated (95% O_2_ and 5% CO_2_) ACSF containing (in mM): 120 NaCl, 2.5 KCl, 1.25 NaH_2_PO_4_, 22 NaHCO_3_, 2 MgSO_4_, 2 CaCl_2_, and 25 glucose (pH 7.35; ∼305 mOsmol/kg). Neurons of the BNST were identified visually by differential interference contrast infrared video microscopy (monochrome-camera CF8/5 NIR, Kappa, Gleichen, Germany) through a 60× water-immersion objective on a confocal microscope (Olympus BX51WI, Olympus, Hamburg, Germany). GFP-positive cells were identified using a 488 nm diode-laser (Coherent, Dieburg, Germany) and confocal laser-scanning-microscopy (Olympus BX51WI, Olympus, Hamburg, Germany). Micropipettes were pulled from borosilicate glass capillaries (GC150TF-10, Harvard Apparatus, Cambridge, United Kingdom) to a resistance of 2–3.5 MΩ, when filled with a potassium-gluconate (K-gluconate) based intracellular solution containing (in mM): 88 K-gluconate, 20 K_3_-citrate, 10 NaCl, 10 HEPES, 3 BAPTA, 15 phosphocreatine, 1 MgCl_2_, 0.5 CaCl_2_, 3 Mg-ATP, and 0.5 Na-GTP (pH 7.25; 290–300 mOsmol/kg). For investigation of GABAergic synaptic activity, a high-chloride intracellular solution was used, containing (in mM): 10 NaCl, 110 KCl, 11 EDTA, 10 HEPES, 1 MgCl_2_, 0.5 CaCl_2_, 15 phosphocreatine, 3 Mg-ATP, and 0.5 Na-GTP (pH 7.25 with KOH; ∼300 mOsmol/kg). Recordings were obtained with a HEKA EPC10 double patch-clamp amplifier (HEKA, Lambrecht/Pfalz, Germany) and PatchMaster recording software (v2.35) at a sampling rate of 10 kHz, with a low pass filter of 2–3 kHz. R_s_ was monitored throughout all experiments. Cells with *R*_s_ > 25 MΩ and an *R*_s_-variance of more than 30% during experiments, were discarded. Basic electrophysiological properties, i.e., RMP or *R*_s_, were noted immediately after whole-cell configuration was established. Cells with RMP positive to −50 mV and APs that did not overshoot +5 mV were discarded.

To determine electrogenic properties, square-wave current pulses from −80 to +100 pA (pulse size varied in 20 pA steps) were injected for a duration of 500 ms in current-clamp mode at −60 mV. R_in_ was calculated from the voltage deflection at the steady state of a −60 pA hyperpolarizing current. Time constant (τ) was calculated from an exponential curve fitting to the hyperpolarizing voltage deflection. *C*_in_ was calculated from τ divided by *R*_in_. Spike properties were assessed using the first AP evoked by injection of +60 pA current steps at −60 mV. Spike threshold was determined from phase-plots. Briefly, the slope of MP (dV/dt) was plotted versus MP. The potential, where the phase-plot slope surpassed 10 mV/ms, was taken as spike threshold (Kress et al., 2009). Spike amplitude was calculated as difference between spike threshold and peak of the AP. Spike half width was then measured as the width of the AP at 50% spike amplitude. For *I*_h_ score, the difference between peak deflection of the MP at the beginning of a −80 pA hyperpolarizing step and the steady state at the end of the 500 ms step was determined, and was divided by the most negative potential at the −80 pA hyperpolarizing step (Sosulina et al., 2010; Daniel et al., 2017). *I*_K(IR)_ score was calculated by dividing the steady state MP at the end of a −20 pA hyperpolarizing step, by the difference between the most hyperpolarizing traces at −80 and −60 pA (Daniel et al., 2017). Early and late adaptation were calculated from instantaneous frequencies of APs at a +60 pA depolarizing step. The instantaneous frequency between the first two APs was deemed *f*_initial_, the instantaneous frequency of the two APs 200 ms into the step was deemed *f*_200_ and the instantaneous frequency of the last two APs was deemed *f*_final_. Early and late adaptation were then calculated as (*f*_initial_–*f*_200_)/*f*_initial_ and (*f*_200_–*f*_final_)/*f*_final_, respectively (Sosulina et al., 2010). Rebound burst firing was assessed from responses to relief of hyperpolarizing current steps (−20 to −80 pA; 500 ms duration; from −60 mV). Classification of cells into three previously described cell types, was done by visual inspection of current-clamp traces from injection square wave current pulses (from −80 to +100 pA) (Hammack et al., 2007; Daniel et al., 2017). Briefly, presence of an *I*_h_ depolarizing sag (>4 mV) was taken to qualify recorded cells as type I. When the presence of the *I*_h_-sag was accompanied by rebound-burst firing behavior, cells were classified as type II. Finally, the absence of *I*_h_-indicating depolarizing sag, and the simultaneous presence of fast inward rectification, qualified cells to be type III (Hammack et al., 2007; Rodríguez-Sierra et al., 2013). Cells that did not show an *I*_h_-dependent depolarizing sag, rebound burst or fast inward rectification were considered unclassified. Recordings used for classification of cells (type I, II, III) were performed without pharmacological manipulation of glutamatergic and GABAergic synaptic transmission.

Synaptic properties were assessed in voltage-clamp mode in the population of GFP-positive and -negative neurons in BNST, and recordings obtained from subclasses of neurons (type I, II, III) were pooled in each population. EPSCs were recorded in presence of GABA receptor blockers (CGP 2.5 μM, gabazine 10 μM) and with a K-gluconate based internal solution, whereas IPSCs were recorded in presence of glutamate receptor blockers (AP-5 20 μM, DNQX 10 μM) and a KCl based internal solution. For measurements of evoked postsynaptic currents, a tungsten bipolar stimulation electrode (0.1 MΩ, 75 μm; MicroProbes, Gaithersburg, MD, United States) was placed dorsally to the recording electrode in the surrounding neuropil and paired events (inter stimulus interval, ISI, 100 ms) were triggered five times for each stimulation intensity (5 μA steps; 10–40 μA) at a rate of 0.1 Hz. Stimulation was considered successful, if events were reliably evoked with latencies shorter than 5 ms and amplitudes exceeding three times RMS of a 100 ms baseline. Response amplitudes were averaged from 5 consecutive pulses at a given stimulation intensity. Spontaneous and miniature postsynaptic currents (s/mIPSCs and s/mEPSCs) were recorded for at least 120 s and cells with less than 30 events (0.25 Hz) were excluded from analysis. For cumulative probability plots, the first 30 events of each experimental group were pooled, and probabilities were calculated for the sum of these grouped events.

Synaptic excitability experiments were conducted in current-clamp mode at a holding potential of −60 mV with no blockers of glutamatergic or GABAergic synaptic transmission. The stimulation intensity needed to evoke a postsynaptic AP was determined with afferent, single pulse electrical stimulation (5 μA steps, starting at 10 μA). When applying stimulation trains (10 pulses at 40 Hz), the stimulus intensity was set to evoke postsynaptic potential amplitudes of less than 70% of the maximum response in response to the first stimulation pulse.

### Morphology

Following electrophysiological characterization, a subset of cells was loaded with 0.2% neurobiotin (Vector Laboratories, Burlingame, CA, United States) by passing +100 pA depolarizing rectangular pulses of 50 ms duration at 10 Hz for 5 to 10 min. After loading, slices were left in the recording chamber for a minimum of 5 min to allow neurobiotin diffusion. For neurobiotin visualization sections were incubated overnight at 4°C in Alexa fluor 546-conjugated streptavidin (1:1,000 in blocking solution; Life Technologies, Eugene, OR, United States). After washing in 0.1 M PBS, slices were mounted on slides and coverslipped with Vectashield HardSet Antifade mounting medium. Fluorescent images were taken using a Nikon D-Eclipse C1 confocal laser scanning microscope equipped with a set of lasers covering the 488 and 543 lines using a Nikon achromatic LWD 16×/0.8w objective. Labeled neurons were reconstructed from z-stacks and total dendritic length and dendritic arbor complexity analyzed using the Simple Neurite Tracer Plugin with its built-in Sholl analysis in Fiji (Longair et al., 2011; Schindelin et al., 2012). Soma size was quantified as soma area measured from z-stack projections. The total number of branches was defined as the sum of the primary, secondary, tertiary, and quaternary processes.

### Cell Harvesting and Single Cell RT-PCR

In order to test for specificity of GFP labeling, a single cell RT-PCR approach as described in Sosulina et al. was used (Sosulina et al., 2008). In brief, the cell content from GFP-positive and GFP-negative neurons was harvested under visual control using a sterile glass electrode filled with 6 μl intracellular solution (described above) by applying negative pressure to the pipette, while keeping the high seal resistance between the pipette tip and the membrane. The intracellular solution containing the cell content was transferred to a microtube filled with 3 μl RNAse free water and stored at −80°C. The RT was performed in a final volume of 20 μl by adding reverse transcriptase buffer (Invitrogen, Karlsruhe, Germany), random hexanucleotide primer (50 μM; Roche, Mannheim, Germany), dNTPs (Thermo-Fisher Scientific, Dreieich, Germany), 20 U RNasin (Promega, Mannheim, Germany), 1 μl of DTT (10 mM), and 0.5 μl of Superscript Reverse Transcriptase III (Invitrogen, Karlsruhe, Germany). After incubation at 37°C for 1 h, cDNA samples were stored at −20°C until use.

A multiplex two round PCR was carried out for the amplification of NPY and the housekeeping gene HPRT, which served as control for successful cell content collection and cDNA synthesis. For the initial multiplex PCR, primers (200 nM each; NPY-forward 5′-CACGATGCTAGGTAACAAG-3′, NPY-reverse 5′-CACATGGAAGGGTCTTCAAG-3′; HPRT-forward 5′-GCAGTCCCAGCGTCGTGA-3′, HPRT-reverse 5′-CAAGGGCATATCCAACAACAAACT-3′; Eurofins Genomics, Ebersberg, Germany), PCR buffer, MgCl_2_ (2.5 mM) and Taq polymerase (1.25 U, Promega, Mannheim, Germany) were added to 10 μl of cDNA sample (RNAse-free H_2_O ad 50 μl). The PCR protocol consisted of an initial denaturation for 5 min at 95°C, 40 cycles of amplification (30 s at 95°C, 30 s at 60°C, 45 s at 72°C) and a final elongation for 5 min at 72°C (Mastercycler gradient, Eppendorf, Hamburg, Germany). PCRs with nested primers for NPY (NPY-nested-forward 5′-ATGGGGCTGTGTGGACTGAC-3′ and NPY-nested-reverse 5′-CTTGTTCTGGGGGCGTTTTC-3′) and HPRT (HPRT-nested-forward 5′-CATTGTGGCCCTCTGTGT-3′ and HPRT-nested-reverse 5′-CAAAGTCTGGCCTGTATCC-3′) were run with 2 μl PCR product from the multiplex PCR as template (PCR protocol as above, but 45 cycles of amplification). Amplified products were run on 1.5% agarose gels and visualized using Midori Green Advance (Biozym, Oldendorf, Germany). Samples with intracellular solution (6 μl) as RT template and H_2_O as PCR template were included as negative controls. Cell samples that were negative for HPRT were excluded from analysis.

### Histology

In order to assess the regional distribution of NPY-positive neurons and localization of recorded neurons in BNST, acute brain slices were placed into 4% PFA in 0.1 M PBS (pH 7.4) and incubated overnight. After about 12 h in PFA, slices were transferred to a 30% Sucrose solution and kept there, until slices were fully immersed. Slices were cut down to 40 μm sections and mounted on superfrost plus microscopy slides (Thermo Fisher Scientific, Waltham, MA United States) with Vectashield HardSet Antifade mounting medium (Vector Laboratories Inc., Burlingame, CA United States). Fixed slices (250–300 μm) containing neurobiotin-filled neurons were washed three times in 0.1 M PBS, then incubated for 3 h at room temperature in a PBS blocking solution containing 10% normal goat serum, 3% bovine serum albumin and 0.3% Triton X-100.

### Blockers/Drugs

All drugs were prepared as high concentrated stock solutions in double-distilled H_2_O (CGP 55845 in dimethyl sulfoxide), stored at −20°C and diluted in ACSF to the final concentration. The following drugs were used: DL-2-amino-5-phosphonopentanoic acid (DL-AP5) sodium salt, 20 μM; 6,7-dinitroquinoxaline-2,3-dione (DNQX) disodium salt, 10 μM; 2-(3-carboxypropyl)-3-amino-6-(4 methoxyphenyl)pyridazinium bromide (SR95531, gabazine), 10 μM; from Abcam (Cambridge, United Kingdom); and (2S)-3-[[(1S)-1-(3,4-dichlorophenyl)ethyl]amino-2-hydroxypropyl](phenylmethyl)phosphinic acid (CGP 55845) hydrochloride, 2.5 μM; octahydro-12-hydroxymethyl-2-imino-5,9:7,10a-dimethano-10aH-[1,3]dioxocino[6,5-d]pyrimidine-4,7,10,11,12-pentol (TTX), 0.5 μM; obtained from Tocris (Wiesbaden-Nordenstadt, Germany).

### Software

Recordings were done with PatchMaster (v. 2.35), converted to ABF-files, and analyzed offline in Clampfit (v. 10.5). Data was collected and averaged in MS Excel 2010; statistics were done in SPSS (v. 24) and GraphPad Prism (v. 6.0). Diagrams from Excel were graphically arranged in CorelDraw (v. 14).

### Statistical Analysis

Each data set was checked for outliers with Grubbs’ test and means ± SEM (standard error of the mean) were calculated. Numbers given in text (x/y) refer to numbers of neurons (x) recorded in different animals (y). Statistical differences between groups and data points were established with independent samples, two-tailed *t*-test, one-way analysis of variance (ANOVA) with *post hoc* Tukey’s multiple comparisons test or two-way RM ANOVA with Holm-Sidak corrected *post hoc* Bonferroni multi comparisons test or *post hoc* LSD test. Differences of distribution of cumulative probabilities were assessed using the Kolmogorov–Smirnov test. Differences in spike probability were assessed with Fisher’s exact test.

## Results

**FIGURE 1:**
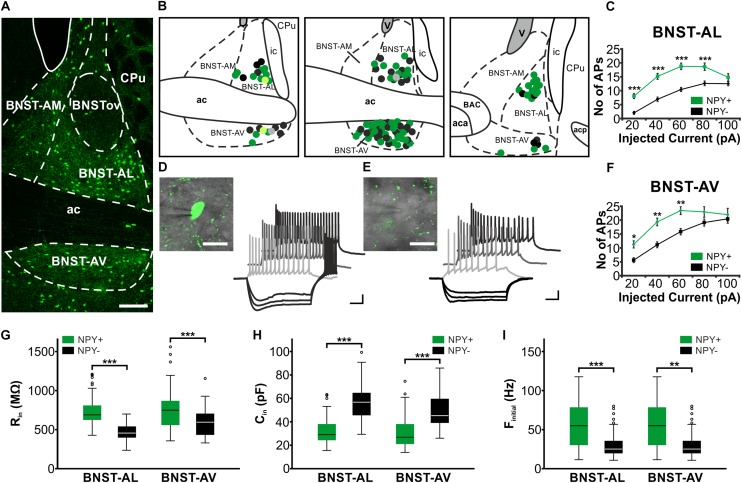
Location and basic properties of NPY-positive and NPY-negative neurons in anterior BNST. **(A)** Pattern of NPY-GFP neurons in anterior BNST, visualized in a confocal image of a coronal acute slice prepared from *npy*-hrGFP mice. Note high densities of GFP-positive cells in BNST-AL and BNST-AV, relatively low densities in BNST-AM, and lack of GFP fluorescence in BNSTov. **(B)** Coordinates of recorded neurons, outlined in schematics of three consecutive coronal sections of the anterior BNST (Bregma: 0.26 mm, 0.14 mm, 0.02 mm). Recorded cells are shown as green (NPY^+^) and black (NPY^−^) symbols. Light green (NPY^+^) and gray (NPY^−^) symbols relate to neurobiotin-filled cells shown in Figure 2. **(C+F)** Plots of number of action potentials (APs) generated at each step of positive current injection (in pA) in BNST-AL (NPY^+^: *n* = 49/17; NPY^−^: *n* = 39/15) and BNST-AV (NPY^+^: *n* = 44/17; NPY^−^: *n* = 33/18) neurons. **(D+E)** Higher magnification and sample traces of current-clamp recordings of a NPY^+^ **(D)** and NPY^−^ **(E)** neuron (see insets) in BNST-AV. Cells were held at –60 mV, and series of de- and hyperpolarizing current steps of 500 ms duration were injected in 20 pA increments from –80 to 100 pA. Note repetitive firing of APs upon membrane depolarization, which occurs at higher frequency at a given current step in the NPY^+^ compared to the NPY^−^ neuron. **(G,H,I)** Box plots of *R*_in_, *C*_in_, and *f*_initial_ for NPY^+^ and NPY^−^ neurons in BNST-AL and BNST-AV, respectively. Scale bars: **A**: 100 μm; **D+E**: 20 μm; 20 mV, 100 ms. ^∗^*p* < 0.05; ^∗∗^*p* < 0.01; ^∗∗∗^*p* < 0.001. ac, anterior commissure; aca, anterior commissure, anterior part; acp, anterior commissure, posterior part; BAC, bed nucleus of the anterior commissure; BNST-AL, anterolateral BNST; BNST-AM, anteromedial BNST; BNST-AV, anteroventral BNST; BNSTov, oval nucleus of the BNST; ic, internal capsule; CPu, caudate putamen (striatum); V, ventricle.

All experiments were performed in acute slices of BNST, prepared from *Npy*-hrGFP transgenic mice (NPY-GFP mice), which reliably show hrGFP-expression in NPY-neurons in various regions of the brain, including arcuate nucleus, dentate gyrus, cortex and striatum, as well as in axons of the PVN, corpus callosum and the olfactory bulb (Partridge et al., 2009; van den Pol et al., 2009). Microscopic analysis of BNST *in vitro* readily revealed GFP-positive and GFP-negative cells in BNST (Figure 1). Furthermore, single cell RT-PCR performed in a subpopulation of recorded BNST neurons revealed the presence of NPY in 14 of 18 GFP-positive neurons, while NPY was lacking in almost all (14 of 15) GFP-negative neurons. Therefore, in the following, GFP-positive and -negative neurons are referred to as NPY^+^ and NPY^−^ neurons, respectively. NPY^+^ neurons were spread throughout BNST-AL and BNST-AV (Figure 1A) with a similar distribution. GFP labeling was also detected in BNST-AM, and almost lacking in the oval nucleus of BNST (BNSTov) (Figure 1A). Hence, in an attempt to characterize the physiological properties of NPY^+^ neurons, we focused on BNST-AL and -AV.

**FIGURE 2:**
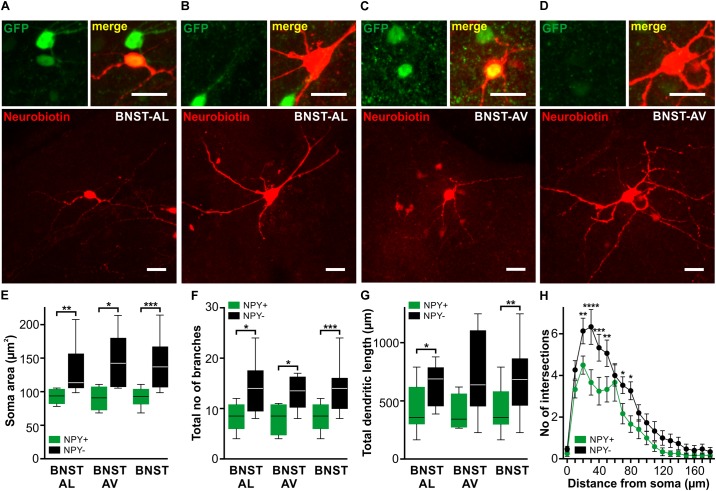
Morphological properties of NPY^+^ and NPY^−^ neurons in anterior BNST. **(A–D)** Confocal z-stack projections showing examples of neurobiotin-filled cells (red channel) in BNST-AL **(A+B)** and BNST-AV **(C+D)**. Upper magnified insets correspond to GFP fluorescence detection on green channel (Left) and the overlay of green and red channels (Right). The merged images clearly identify NPY^+^ **(A+C)** and NPY^−^ **(B+D)** cells by overlap of GFP fluorescence and neurobiotin-filling or lack of GFP fluorescence in filled cells. Scale bars in panels **(A–D)**: 20 μm. Please, refer to Figure 1B for localization of neurobiotin-filled cells. **(E–G)** Box plots of soma area **(E)**, total number of branches **(F)** and total dendritic length **(G)** in NPY^+^ and NPY^−^ neurons reveal smaller somata and shorter, less ramified dendritic tree in NPY^+^ (BNST-AL: *n* = 8/3, BNST-AV: *n* = 4/3) compared to NPY^−^ neurons (BNST-AL: *n* = 9/7, BNST-AV: *n* = 6/4, unpaired *t*-test, ^∗^*p* < 0.05, ^∗∗^*p* < 0.01, ^∗∗∗^*p* < 0.001). **(H)** Number of intersecting branches plotted against distance from center of the soma comparing NPY^+^ and NPY^−^ neurons [^∗^*p* < 0.05, ^∗∗^*p* < 0.01, ^∗∗∗^*p* < 0.001, ^∗∗∗∗^*p* < 0.0001 by *post hoc* LSD test on two-way RM ANOVA, factor NPY: *F*_(1,25)_ = 7.95, *p* < 0.01].

### NPY^+^ Neurons in Anterior BNST Display Fast Spiking Activity

Single visually identified NPY^+^ and NPY^−^ neurons were recorded using whole-cell patch-clamp recordings in BNST-AL (NPY^+^: *n* = 49/17; NPY^−^: *n* = 39/15) and BNST-AV (NPY^+^: *n* = 44/17; NPY^−^: *n* = 33/18), with recording sites verified according to the Paxinos mouse atlas (Paxinos and Franklin, 2001; Figure 1B). Basic electrophysiological membrane properties did not significantly differ in neurons recorded from male (*n* = 53) and female (*n* = 112) mice in these regions, and data were pooled in the following.

While the RMP was not different between types of neurons (BNST-AL: NPY^+^ = −58.8 ± 0.9 mV, NPY^−^ = −59.4 ± 0.8 mV, unpaired *t*-test: *p* = 0.64; BNST-AV: NPY^+^ = −62.1 ± 1.4 mV, NPY^−^ = −63.0 ± 1.2 mV, unpaired *t*-test: *p* = 0.61), the *R*_in_ was significantly higher in NPY^+^ as compared to NPY^−^ neurons in both BNST-AL and -AV (BNST-AL: NPY^+^ = 743.3 ± 28.3 MΩ, NPY^−^ = 458.0 ± 18.4 MΩ, unpaired *t*-test: *p* < 0.001; BNST-AV: NPY^+^ = 776.7 ± 39.9 MΩ, NPY^−^ = 591.1 ± 32.1 MΩ, unpaired *t*-test: *p* < 0.001) (Figure 1G). Furthermore, the *C*_in_ was significantly lower in NPY^+^ compared to NPY^−^ neurons (BNST-AL: NPY^+^ = 32.0 ± 1.7 pF, NPY^−^ = 57.6 ± 2.4 pF, unpaired *t*-test: *p* < 0.001; BNST-AV: NPY^+^ = 30.8 ± 2.1 pF, NPY^−^ = 48.5 ± 2.7 pF, unpaired *t*-test: *p* < 0.001) (Figure 1H). In order to investigate electrogenic membrane properties, neurons were held at −60 mV, and series of depolarizing and hyperpolarizing current steps (duration 500 ms; 20 pA increases in step size) were injected under current-clamp conditions. Both types of neurons in the two regions of BNST responded to injection of depolarizing current steps with series of APs, whose frequencies increased with increasing current strength (Figures 1D,E). An apparent difference was the relatively high frequency of firing observed in NPY^+^ neurons in comparison to NPY^−^ neurons. In fact, in both BNST-AL and BNST-AV, the number of APs generated in response to a current step at a given amplitude was significantly higher in NPY^+^ than NPY^−^ neurons (Figures 1C,F). RM ANOVA revealed a significant difference in spike firing across all tested current injections between the two types of neurons (BNST-AL: *F*_1,86_ = 24.26; *p* < 0.001; BNST-AV: *F*_1,75_ = 7.795; *p* < 0.01), which was corroborated for single current steps by a Holm-Sidak corrected *post hoc* Bonferroni multi comparison test (Figures 1C,F). This difference was also seen in instantaneous firing frequencies (calculated from the first two spikes at +60 pA), which were higher in NPY^+^ neurons than in their NPY^−^ counterparts (BNST-AL: NPY^+^ = 56.6 ± 4.0 Hz, NPY^−^ = 31.4 ± 2.9 Hz, unpaired *t*-test: *p* < 0.001; BNST-AV: NPY^+^ = 80.0 ± 4.8 Hz, NPY^−^ = 57.6 ± 6.1 Hz, unpaired *t*-test: *p* < 0.01) (Figures 1I). Spike properties (spike threshold, spike amplitude, and spike half-width) did not differ between NPY^+^ and NPY^−^ neurons, except for a difference in half width in BNST-AV (NPY^+^ = 0.75 ± 0.03 ms, NPY^−^ = 0.85 ± 0.04 ms, unpaired *t*-test: *p* < 0.05) (Table 2). When comparing the NPY^+^ neuronal subpopulation between BNST-AL and -AV regions, we found a significantly more positive RMP and an increased spike half width in NPY^+^ neurons in BNST-AL (RMP: unpaired *t*-test: *p* < 0.05; spike half width: unpaired *t*-test: *p* < 0.001). Spike amplitude, *I*_h_ score and *f*_initial_ were significantly decreased in NPY^+^ neurons in BNST-AL, when comparing them to NPY^+^ neurons in BNST-AV (spike amplitude: unpaired *t*-test: *p* < 0.001; *I*_h_ score: unpaired *t*-test: *p* < 0.05; *f*_initial_: unpaired *t*-test: *p* < 0.001).

To study the morphological properties of recorded cells, a subset of cells was filled with neurobiotin. In total, 27 neurobiotin-filled cells were recovered from BNST-AL (NPY^+^: *n* = 8/3; NPY^−^: *n* = 9/7) and BNST-AV (NPY^+^: *n* = 4/3; NPY^−^: *n* = 6/4). Examples are illustrated in Figure 2. Basic electrotonic cellular properties (RMP, *R*_in_, and *C*_in_) and instantaneous firing frequencies in this subset of cells matched the larger sample displayed in Figure 1 and Tables 1, 2 (RMP: BNST-AL: NPY^+^ = −56.6 ± 2.1 mV, NPY^−^ = −60.8 ± 2.6 mV, unpaired *t*-test: *p* = 0.27; BNST-AV: NPY^+^ = −59.0 ± 2.7 mV, NPY^−^ = −59.3 ± 3.4 mV, unpaired *t*-test: *p* = 0.95; *R*_in_: BNST-AL: NPY^+^ = 728.7 ± 79.4 MΩ, NPY^−^ = 467.8 ± 37.7 MΩ, unpaired *t*-test: *p* < 0.01; BNST-AV: NPY^+^ = 810.6 ± 84.5 MΩ, NPY^−^ = 396.9 ± 23.7 MΩ, unpaired *t*-test: *p* < 0.01; *C*_in_: BNST-AL: NPY^+^ = 31.1 ± 3.4 pF, NPY^−^ = 56.0 ± 6.1 pF, unpaired *t*-test: *p* < 0.01; BNST-AV: NPY^+^ = 25.8 ± 4.0 pF, NPY^−^ = 63.7 ± 4.8 pF, unpaired *t*-test: *p* < 0.001; *f*_initial_: BNST-AL: NPY^+^ = 59.7 ± 5.0 Hz, NPY^−^ = 31.7 ± 5.0 Hz, unpaired *t*-test: *p* < 0.01; BNST-AV: NPY^+^ = 82.3 ± 11.2 Hz, NPY^−^ = 22.0 ± 5.4 Hz, unpaired *t*-test: *p* < 0.01). Confocal microscopic imaging of neurobiotin-filled cells indicated smaller somata and overall less ramified dendritic trees in NPY^+^ compared to NPY^−^ neurons in anterior BNST (Figures 2A–D), corresponding to higher *R*_in_ and lower *C*_in_ recorded in NPY^+^ neurons. In fact, somata of NPY^+^ neurons were smaller than those of NPY^−^ neurons (BNST-AL: NPY^+^ = 93.0 ± 3.7 μm^2^, NPY^−^ = 132.7 ± 12.0 μm^2^, unpaired *t*-test: *p* < 0.01, BNST-AV: NPY^+^ = 90.3 ± 8.9 μm^2^, NPY^−^ = 147.1 ± 16.7 μm^2^, unpaired *t*-test: *p* < 0.05, Figure 2E). Furthermore, NPY^+^ neurons had lower total number of processes (BNST-AL: NPY^+^ = 8.25 ± 0.98, NPY^−^ = 14.22 ± 1.70, unpaired *t*-test: *p* < 0.05, BNST-AV: NPY^+^ = 8.00 ± 1.58, NPY^−^ = 13.71 ± 1.35, unpaired *t*-test: *p* < 0.05, Figure 2F) and lower total dendritic length in BNST-AL (BNST-AL: NPY^+^ = 426.6 ± 73.3 μm, NPY^−^ = 635.9 ± 58.9 μm, unpaired *t*-test: *p* < 0.05, BNST-AV: NPY^+^ = 392.1 ± 79.2 μm, NPY^−^ = 723.2 ± 151.4 μm, unpaired *t*-test: *p* = 0.14, Figure 2G). As we found no differences in these morphological properties between BNST-AL and BNST-AV (soma area: *p* = 0.74 for NPY^+^ and *p* = 0.48 for NPY^−^, unpaired *t*-test; total number of processes: *p* = 0.89 for NPY^+^ and *p* = 0.66, unpaired *t*-test; total dendritic length: *p* = 0.78 for NPY^+^ and *p* = 0.55 for NPY^−^, unpaired *t*-test), neurons from both BNST regions were pooled for quantification of the number of intersecting branches at increasing distance from center of soma (radius step size 10 μm). NPY^+^ neurons showed less complex branching patterns compared to NPY^−^ neurons (Figure 2H). Notably, the majority of neurons bore dendritic varicosities.

**Table 1:** Intrinsic membrane properties of NPY^+^ and NPY^−^ neurons in BNST-AL.

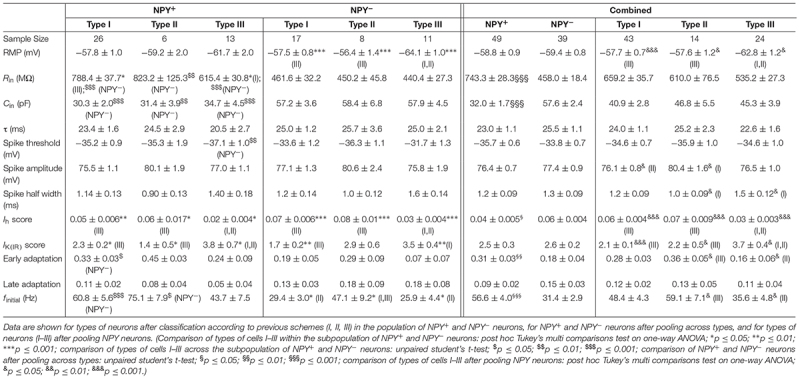

**Table 2:** Intrinsic membrane properties of NPY^+^ and NPY^−^ neurons in BNST-AV.

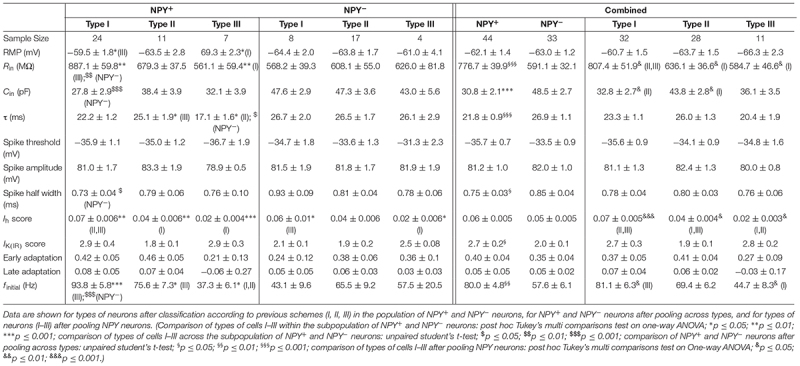

### NPY^+^ Neurons Comprise Various Classes of Neurons in Anterior BNST

**FIGURE 3:**
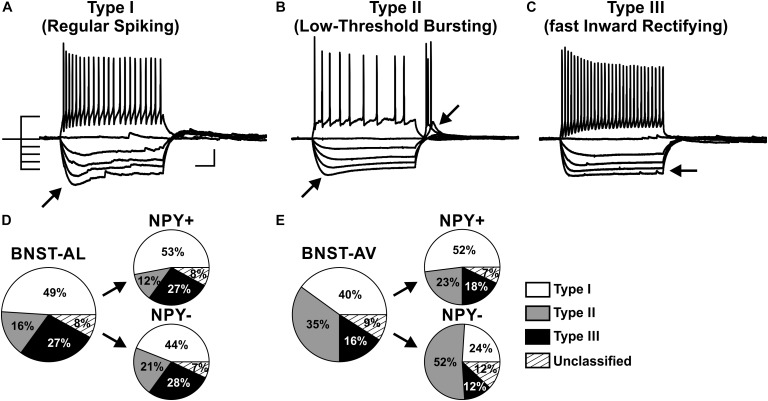
Proportion of three physiological phenotypes in NPY^+^ and NPY^−^ neurons in anterior BNST. **(A–C)** Example traces of current-clamp recordings from BNST-AL neurons classified as type I (RS), type II (LTB), and type III (fIR). Exemplary traces obtained in response to a depolarizing current step (60 pA) and a series of hyperpolarizing current steps (−80 to 0 pA) from –60 mV are shown. **(D)** Proportion of cell types I–III recorded in BNST-AL, overall (large pie chart; *n* = 88/23) and separate for NPY^+^ (*n* = 49/17) and NPY^−^ (*n* = 39/15) neurons. **(E)** Proportion of cell types I–III recorded in BNST-AV, overall (*n* = 77/22) and separate for NPY^+^ (*n* = 44/17) and NPY^−^ (*n* = 33/18) neurons. Scale bar in panels **(A–C)**: 20 mV, 100 ms.

A widely accepted classification scheme distinguishes between three major types of neurons in BNST-AL mostly based on electrophysiological properties (Hammack et al., 2007; Rodríguez-Sierra et al., 2013; Daniel et al., 2017). According to this scheme, type I neurons (Regular Spiking, RS) are regular spiking, while type 2 neurons (Low-Threshold Bursting, LTB) generate a low-threshold burst of APs due to activation of an *I*_T_ Ca^2+^-current, which is typically observed as a rebound spike burst upon relief of membrane hyperpolarization. Both types of neurons display anomalous inward rectification apparent as a slowly developing depolarizing sag upon maintained membrane hyperpolarization due to activation of an *I*_h_ current (arrows Figures 3A,B). Both types of neurons can thus be distinguished from type III neurons (fast Inward Rectifying, fIR) displaying fast inward rectification in the hyperpolarizing direction due to activation of a *I*_K(IR)_ current (arrow in Figure 3C). These major types of neurons could be readily discerned in the population of neurons recorded in the present study (*n* = 165). Examples are illustrated in Figures 3A–C, quantitative data are shown in Table 1 (BNST-AL) and Table 2 (BNST-AV). All three types of neurons were encountered in BNST-AL and BNST-AV, and only a minority of cells (9 and 8%) did not fall into these categories. In BNST-AL, type I, II, and III neurons comprised 49, 16, and 27% of recorded neurons, respectively (Figure 3D). A similar proportion of the three electrophysiological cell types was observed in NPY^+^ and NPY^−^ neurons (53%, 12%, 27% versus 44%, 21%, 28%; Figure 3D). Furthermore, in the NPY^+^ subpopulation, *R*_in_ was significantly lower in type III neurons, compared to type I (type I: 788.4 ± 37.7 MΩ, type III: 615.4 ± 30.8 MΩ; *post hoc* Tukey’s multi comparisons test, *p* < 0.05; Table 1). In conformity with classification parameters, *I*_h_ score was significantly lower and *I*_K(IR)_ score was significantly higher in type III neurons, compared to type I and type II (Table 1). In the NPY^−^ subpopulation of BNST-AL neurons, RMP was significantly more negative in type III neurons, compared to type I and type II neurons. Finally, *f*_initial_ was significantly increased in type II NPY^−^ BNST-AL neurons, compared to type I and type III (Table 1). In BNST-AV, type I, II, and III contributed 40, 35, 16% to recorded neurons, and distinction between the NPY^+^ and NPY^−^ subpopulation revealed a relative preponderance of type I NPY^+^ (52%) and type II NPY^−^ (52%) neurons (Figure 3E). Here, in the NPY^+^ subpopulation, type III neurons differed significantly from type I neurons in terms of RMP, *R*_in_, *I*_h_ score and *f*_initial_, whereas significant differences from type II neurons were apparent in τ and *f*_initial_ (Table 2). In the NPY^−^ subpopulation, *I*_h_ score was significantly different between type I and type III neurons (Table 2).

### Properties of Excitatory Synaptic Inputs to NPY^+^ and NPY^−^ Neurons in Anterior BNST

**FIGURE 4:**
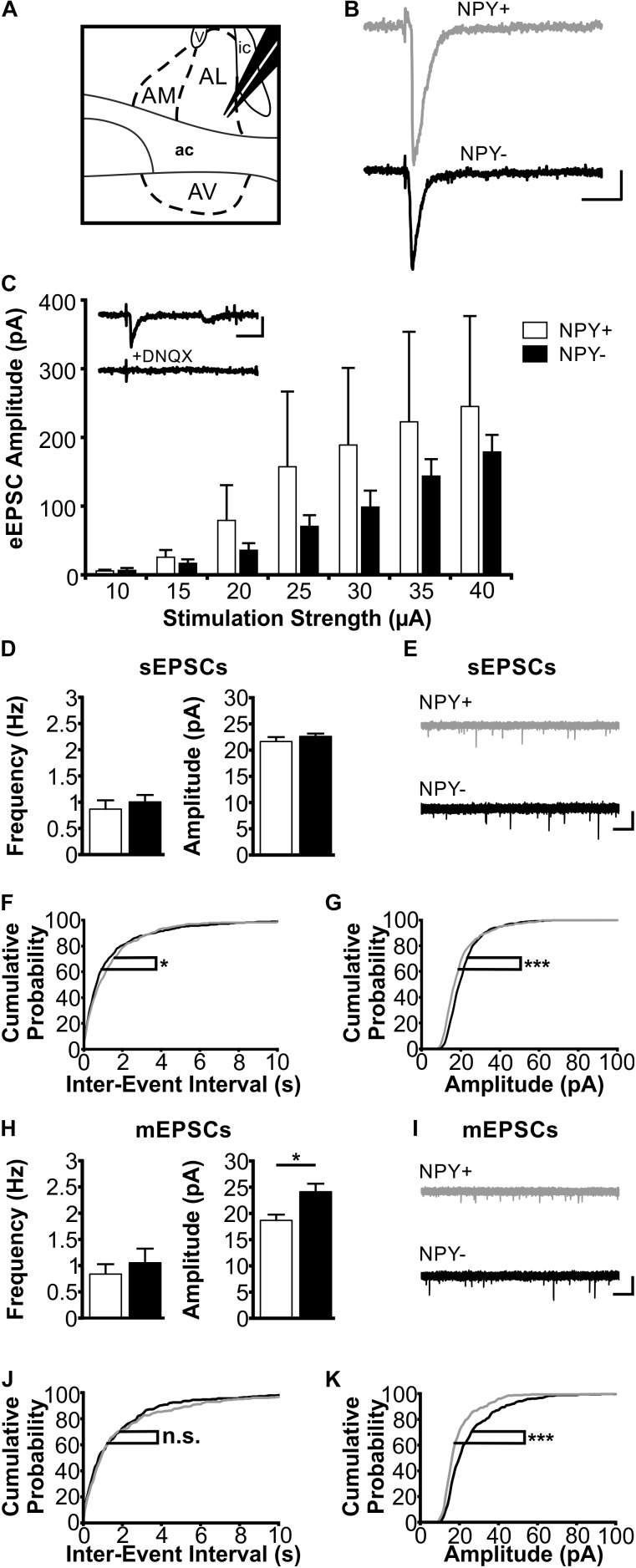
Excitatory postsynaptic responses of NPY^+^ and NPY^−^ neurons in BNST-AL. **(A)** Schematic illustration of a coronal BNST section, showing placement of the stimulation electrode in BNST-AL. **(B)** Exemplary traces of an eEPSC recorded in a NPY^+^ (Top) and a NPY^−^ (Bottom) neuron at a stimulation intensity of 30 μA. **(C)** Plot of eEPSC amplitudes of NPY^+^ and NPY^−^ neurons at stimulation intensities from 10 to 40 μA (5 μA increments) (NPY^+^: *n* = 6/5; NPY^−^: *n* = 7/6). Inset: example of DNQX (10 μM) sensitivity (of eEPSCs in a NPY^+^ neuron. **(D)** Averages of sEPSC frequencies and amplitudes in NPY^+^ (*n* = 18/9; open bars) and NPY^−^ (*n* = 17/8; closed bars) neurons in BNST-AL. **(E)** Corresponding example traces of sEPSCs. **(F+G)** Cumulative probability plots for sEPSC frequencies and amplitudes in BNST-AL. Both show a significantly different distribution in a Kolmogorov–Smirnov test (frequencies: ^∗^*p* < 0.05; amplitudes: ^∗∗∗^*p* < 0.001) (NPY^+^ events: *n* = 540; NPY^−^ events: *n* = 510). **(H)** Averages of mEPSC frequencies and amplitudes recorded in NPY^+^ (*n* = 11/8; open bars) and NPY^−^ (*n* = 12/7; closed bars) neurons in BNST-AL. mEPSC amplitudes are significantly smaller in NPY^+^ neurons (unpaired *t*-test; ^∗^*p* < 0.05). **(I)** Corresponding example traces of mEPSCs (NPY^+^: gray; NPY^−^: black). **(J+K)** Cumulative probability plots for mEPSC frequencies and amplitudes. Only amplitudes show a significantly different distribution in a Kolmogorov–Smirnov test (frequencies: *p* = 0.32; amplitudes: ^∗∗∗^*p* < 0.001) (NPY^+^ events: *n* = 330; NPY^−^ events: *n* = 360). Scale bars: **B+C**: 20 ms and 20 pA; **E+I**: 1 s and 20 pA.)

**FIGURE 5:**
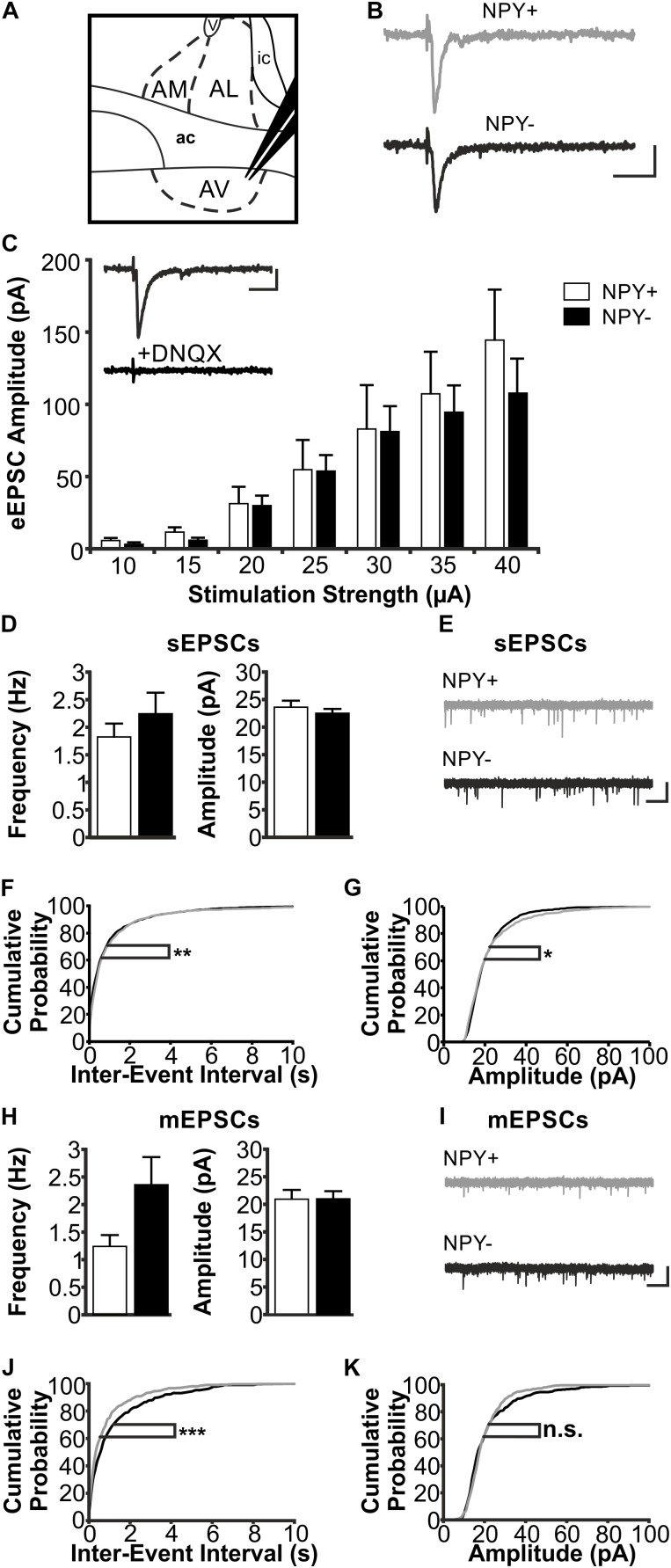
Excitatory postsynaptic responses of NPY^+^ and NPY^−^ neurons in BNST-AV. **(A)** Schematic illustration of a coronal BNST section, showing placement of the stimulation electrode in BNST-AV. **(B)** Exemplary traces of an eEPSC recorded in a NPY^+^ (Top) and a NPY^−^ (Bottom) neuron at a stimulation intensity of 30 μA. **(C)** Plot of eEPSC amplitudes of NPY^+^ and NPY^−^ neurons at stimulation intensities from 10 to 40 μA (5 μA increments) (NPY^+^: *n* = 14/7; NPY^−^: *n* = 13/7). Inset: DNQX (10 μM) sensitivity of eEPSC in a NPY^−^ neuron. **(D)** Averages of sEPSC frequencies and amplitudes in NPY^+^ (39/17; open bars) and NPY^−^ (*n* = 34/17; closed bars) neurons recorded in BNST-AV. **(E)** Corresponding example traces of sEPSCs (NPY^+^: gray; NPY^−^: black). **(F+G)** Cumulative probability plots for sEPSC frequencies and amplitudes in BNST-AV. Both show a significantly different distribution in a Kolmogorov–Smirnov test (frequencies: ^∗∗^*p* < 0.01; amplitudes: ^∗^*p* < 0.05) (NPY^+^ events: *n* = 1170; NPY^−^ events: *n* = 1020). **(H)** Averages of mEPSC frequencies and amplitudes recorded in NPY^+^ (*n* = 14/9; open bars) and NPY^−^ (*n* = 17/10; closed bars) neurons in BNST-AV. **(I)** Corresponding example traces of mEPSCs (NPY^+^: gray; NPY^−^: black). **(J+K)** Cumulative probability plots for mEPSC frequencies and amplitudes. Only frequencies show a significantly different distribution in a Kolmogorov–Smirnov test (frequencies: ^∗∗∗^*p* = 0.001; amplitudes: *p* = 0.12) (NPY^+^ events: *n* = 420; NPY^−^ events: *n* = 510). Scale bars: **B+C**: 20 ms and 20 pA; **E+I**: 1 s and 20 pA.)

In a next experimental step, we compared synaptic properties between NPY^+^ and NPY^−^ neurons in BNST. First, we focused on excitatory synaptic responses, recorded in single neurons under voltage-clamp conditions during blocked GABAergic transmission (see section “Materials and Methods” for further details). A stimulation electrode was placed locally in the neuropil dorsal to the recording site in BNST-AL or -AV to evoke postsynaptic responses (Figures 4A, 5A). The short latency (<5 ms) with little variation and the smooth trajectory of evoked synaptic responses indicated that they were monosynaptic in nature (Figures 4B, 5B), and blockade by DNQX (10 μM) indicated mediation by AMPA receptors (Figures 4C, 5C).

In BNST-AL, amplitudes of eEPSCs increased with increasing strength of the stimulation current (range 10–40 μA), and significant differences were not observed between NPY^+^ (*n* = 6/5) and NPY^−^ neurons (*n* = 7/6) (Figure 4C). Furthermore, spontaneous EPSCs (sEPSCs) were observed in BNST-AL NPY^+^ (*n* = 18/9) and NPY^−^ (*n* = 17/8) neurons, with no apparent differences in average frequency (NPY^+^ = 0.87 ± 0.17 Hz, NPY^−^ = 1.0 ± 0.13 Hz, unpaired *t*-test, *p* = 0.55) or amplitude (NPY^+^ = 21.7 ± 0.8 pA, NPY^−^ = 22.6 ± 0.6 pA, unpaired *t*-test, *p* = 0.37) between types of cells (Figures 4D,E). Cumulative probability plots of sEPSCs revealed a different distribution of amplitudes and frequencies in the two populations of neurons (Kolmogorov–Smirnov test; frequency: *p* < 0.05; amplitude: *p* < 0.001; NPY^+^ events: *n* = 540; NPY^−^ events: *n* = 510) (Figures 4F,G). Finally, miniatures EPSCs (mEPSCs) were analyzed in the presence of TTX (0.5 μM), in NPY^+^ (*n* = 11/8) and NPY^−^ (*n* = 12/7) neurons. While there was no difference in average frequency between neurons (NPY^+^ = 0.83 ± 0.19 Hz; NPY^−^ = 1.05 ± 0.27 Hz; unpaired *t*-test: *p* = 0.54), the average amplitude of mEPSCs was significantly smaller in NPY^+^ (18.7 ± 1.1 pA) than in NPY^−^ neurons (24.1 ± 1.5 Hz; unpaired *t*-test: *p* < 0.05) (Figures 4H,I). Cumulative probability plots corroborated this difference in mEPSC amplitude but not frequencies between types of neurons. (Kolmogorov–Smirnov test, *p* < 0.001; NPY^+^, 330 events; NPY^−^, 360 events) (Figures 4J,K).

In BNST-AV, differences between NPY^+^ and NPY^−^ neurons were not apparent from recordings of eEPSCs (NPY^+^: *n* = 14/7; NPY^−^: *n* = 13/7) (Figure 5C) nor sEPSCs (NPY^+^: *n* = 39/17; NPY^−^: *n* = 34/17; frequency: NPY^+^ = 1.82 ± 0.24 Hz, NPY^−^ = 2.24 ± 0.38 Hz, unpaired *t*-test, *p* = 0.46; amplitude: NPY^+^ = 23.6 ± 1.2 pA, NPY^−^ = 22.5 ± 0.8 pA, unpaired *t*-test, *p* = 0.35) (Figures 5D,E). However, there were differences in distribution of both amplitudes and frequencies of sEPSCs between the two populations of neurons, as revealed by cumulative probability plots (Kolmogorov–Smirnov test; frequency: *p* < 0.01; amplitude: *p* < 0.05; NPY^+^ events: *n* = 1170; NPY^−^ events: *n* = 1020) (Figures 5F,G). Averaging amplitude and frequency of mEPSCs yielded no differences between types of neurons (NPY^+^: *n* = 14/9; NPY^−^: *n* = 17/10; frequency: NPY^+^ = 1.24 ± 0.21 Hz, NPY^−^ = 2.36 ± 0.50 Hz, unpaired *t*-test, *p* = 0.07; amplitude: NPY^+^ = 20.9 ± 1.7 pA, NPY^−^ = 21.0 ± 1.4 pA, unpaired *t*-test, *p* = 0.97) (Figures 5H,I), although cumulative probability plots revealed a significant difference in frequency distribution (Kolmogorov–Smirnov test *p* < 0.001; NPY^+^, 420 events; NPY^−^, 510 events) (Figures 5J,K).

### Properties of Inhibitory Synaptic Inputs to NPY^+^ and NPY^−^ Neurons in Anterior BNST

**FIGURE 6:**
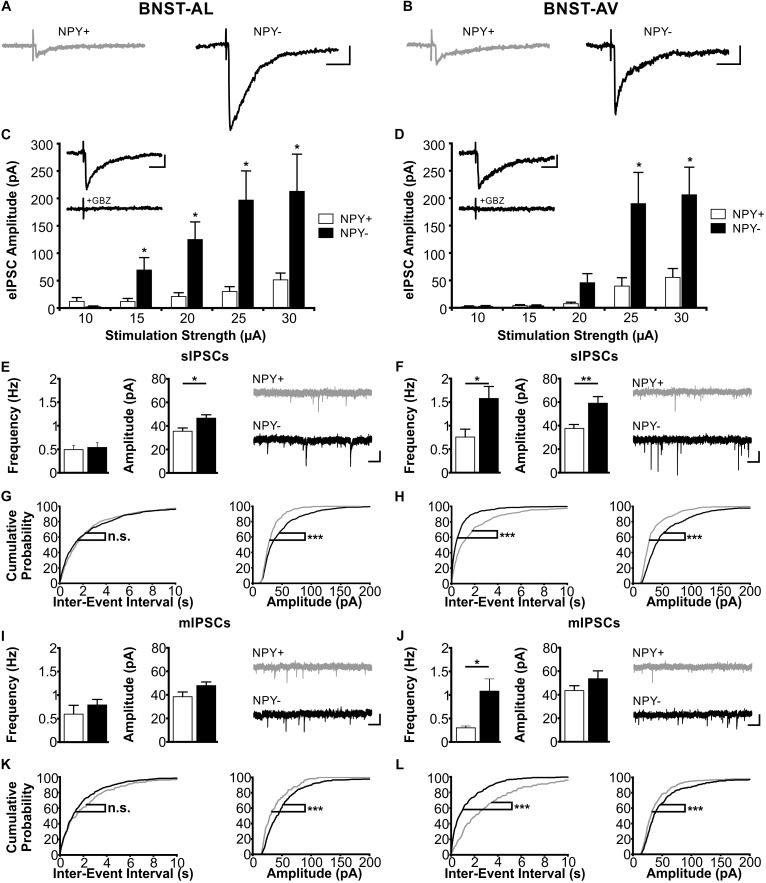
Inhibitory synaptic properties in NPY^+^ and NPY^−^ neurons in BNST-AL (Left column) and BNST-AV (Right column). **(A+B)** Exemplary traces of eIPSCs recorded in NPY^+^ (gray) and NPY^−^ (black) neurons in BNST-AL **(A)** and -AV **(B)** at a stimulation intensity of 25 μA. **(C+D)** Plot of eIPSC amplitudes of NPY^+^ and NPY^−^ neurons at stimulation intensities from 10 to 30 μA (5 μA increments) in BNST-AL (**C**; NPY^+^, *n* = 8/5; NPY^−^, *n* = 8/5) and BNST-AV (**D**; NPY^+^, *n* = 10/6; NPY^−^, *n* = 13/10). eIPSC amplitudes in NPY^+^ neurons are significantly decreased (unpaired *t*-test, ^∗^*p* < 0.05). Insets: gabazine (GBZ, 10 μM) sensitivity of eIPSC in NPY^+^ neurons. **(E)** Averages of sIPSC frequencies and amplitudes in NPY^+^ (16/9; open bars) and NPY^−^ (*n* = 24/10; closed bars) neurons recorded in BNST-AL, with corresponding example traces of sIPSCs (NPY^+^: gray; NPY^−^: black). sIPSC amplitudes are significantly smaller in NPY^+^ neurons (unpaired *t*-test; ^∗^*p* < 0.05). **(F)** Averages of sIPSC frequencies and amplitudes in NPY^+^ (15/8; open bars) and NPY^−^ (*n* = 19/11; closed bars) neurons recorded in BNST-AV, with corresponding example traces of sIPSCs (NPY^+^: gray; NPY^−^: black). sIPSC frequencies (unpaired *t*-test; ^∗^*p* < 0.05) and amplitudes (unpaired *t*-test: ^∗∗^*p* < 0.01) are significantly smaller in NPY^+^ neurons. **(G+H)** Cumulative probability plots for sIPSC frequencies and amplitudes in BNST-AL and -AV. Amplitudes in both regions, and frequencies in BNST-AV show a significantly different distribution in a Kolmogorov–Smirnov test (^∗∗∗^*p* < 0.001) (BNST-AL: NPY^+^ events: *n* = 480; NPY^−^ events: *n* = 720; BNST-AV: NPY^+^ events: *n* = 450; NPY^−^ events: *n* = 570). **(I)** Averages of mIPSC frequencies and amplitudes recorded in NPY^+^ (*n* = 6/5; open bars) and NPY^−^ (*n* = 13/8; closed bars) neurons in BNST-AL, with corresponding example traces of mIPSCs. **(J)** Averages of mIPSC frequencies and amplitudes recorded in NPY^+^ (*n* = 8/6; open bars) and NPY^−^ (*n* = 11/7; closed bars) neurons in BNST-AV, with corresponding example traces of mIPSCs. mIPSC frequencies are significantly smaller in NPY^+^ neurons (unpaired *t*-test; ^∗^*p* < 0.05). **(K+L)** Cumulative probability plots for mIPSC frequencies and amplitudes in BNST-AL and -AV. Amplitudes in both regions, and frequencies in BNST-AV show a significantly different distribution in a Kolmogorov–Smirnov test (^∗∗∗^*p* < 0.001) (BNST-AL: NPY^+^ events: *n* = 180; NPY^−^ events: *n* = 390; BNST-AV: NPY^+^ events: *n* = 240; NPY^−^ events: *n* = 330). Scale bars example traces **A–D**: 1 s and 20 pA. Scale bars **E+F+I+J**: 20 ms and 50 pA.

Next, we focused on inhibitory synaptic responses, recorded in single neurons in BNST-AL and -AV under voltage-clamp conditions in the absence of glutamatergic transmission (see section “Materials and Methods” for further details). Blockade of synaptic events by gabazine indicated mediation by GABA_A_ receptors (Figures 6C,D insets). The experimental scheme followed that described for EPSCs above. Significant differences were found in IPSC properties between NPY^+^ and NPY^−^ neurons in both BNST-AL and -AV (Figure 6). Evoked IPSCs were significantly smaller in amplitude in NPY^+^ neurons than in NPY^−^ neurons over a wide range of tested stimulation intensities, as recorded in BNST-AL (NPY^+^, *n* = 8/5; NPY^−^, *n* = 8/5) and BNST-AV (NPY^+^, *n* = 10/6; NPY^−^, *n* = 13/10) (Figures 6C,D). This cell-type specific difference was also observed in sIPSCs, in that average amplitudes were significantly smaller in NPY^+^ neurons than in NPY^−^ neurons (BNST-AL, NPY^+^: *n* = 16/9: 35.5 ± 2.8 pA; NPY^−^: *n* = 24/10: 46.6 ± 2.9 pA, unpaired *t*-test: *p* < 0.05 BNST-AV, NPY^+^: *n* = 15/8: 37.7 ± 3.3 pA; NPY^−^, *n* = 19/11: 59.0 ± 5.7 pA, unpaired *t*-test: *p* < 0.01) (Figures 6E,F). Frequencies of sIPSCs were not different between cells in BNST-AL (NPY^+^ = 0.49 ± 0.09 Hz, NPY^−^ = 0.54 ± 0.10 Hz, unpaired *t*-test: *p* = 0.76), but decreased in NPY^+^ neurons in BNST-AV (NPY^+^ = 0.76 ± 0.17 Hz, NPY^−^ = 1.58 ± 0.26 Hz, unpaired *t*-test: *p* < 0.05) (Figures 6E,F). These significant differences of sIPSCs in BNST-AL and -AV were also represented in the distribution of cumulative probability plots (Kolmogorov–Smirnov test: *p* < 0.001, BNST-AL: NPY^+^ events: *n* = 480; NPY^−^ events: *n* = 720; BNST-AV: NPY^+^ events: *n* = 450; NPY^−^ events: *n* = 570) (Figures 6G,H). Furthermore, these cell-type specific characteristics were also reflected in mIPSC properties, displaying differences between NPY^+^ and NPY^−^ neurons in amplitude distribution in BNST-AL and BNST-AV, and in frequency distribution in BNST-AV (BNST-AL: NPY^+^, *n* = 6/5, 180 events; NPY^−^, *n* = 13/8, 390 events; Kolmogorov–Smirnov test, frequency: *p* = 0.99, amplitude: *p* < 0.001; BNST-AV: NPY^+^: *n* = 8/6, 240 events; NPY^−^: *n* = 11/7, 330 events; frequency: *p* < 0.001, amplitude: *p* < 0.001) (Figures 6K,L). Finally, mIPSC frequencies were on average smaller in NPY^+^ than in NPY^−^ neurons in BNST-AV (0.30 ± 0.04 Hz versus 1.08 ± 0.26 Hz, unpaired *t*-test: *p* < 0.05), while averaging mIPSCs yielded no further differences between cells (BNST-AL: frequency, NPY^+^ = 0.59 ± 0.19 Hz, NPY^−^ = 0.64 ± 0.12 Hz, unpaired *t*-test: *p* = 0.86; amplitude, NPY^+^ = 39.0 ± 2.8 pA, NPY^−^ = 48.6 ± 3.4 pA, unpaired *t*-test: *p* = 0.11; BNST-AV: amplitude, NPY^+^ = 43.4 ± 4.2 pA, NPY^−^ = 53.6 ± 6.7 pA, unpaired *t*-test: *p* = 0.28) (Figures 6I,J).

### Synaptic Excitability of NPY^+^ and NPY^−^ Neurons in Anterior BNST

**FIGURE 7:**
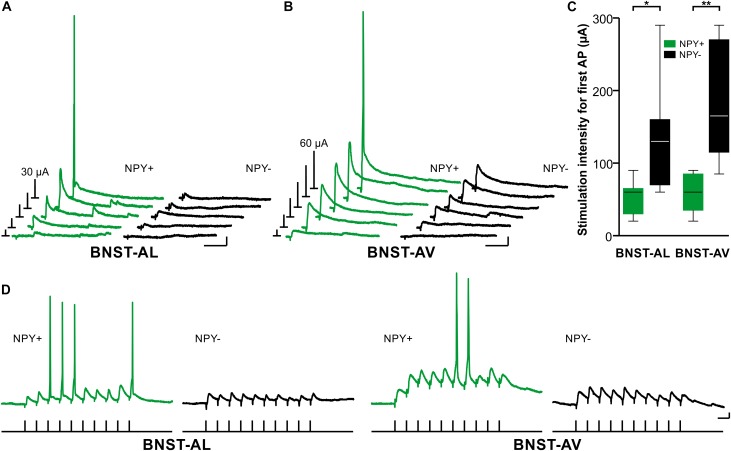
Synaptic excitability of NPY^+^ and NPY^−^ neurons in anterior BNST. **(A+B)** Exemplary traces of representative NPY^+^ and NPY^−^ neurons showing postsynaptic potentials in response to increasing afferent electrical stimulation in BNST-AL (starting from 10 μA in 5 μA steps) and BNST-AV (starting from 10 μA in 10 μA steps). Bars on the left in each panel represent stimulation intensity (in μA, as indicated). **(C)** Box plots of stimulation intensity needed to evoke a spike in NPY^+^ and NPY^−^ neurons reveal higher synaptic excitability in NPY^+^ compared to NPY^−^ neurons (unpaired *t*-test, ^∗^*p* < 0.05, ^∗∗^*p* < 0.01). **(D)** Exemplary traces of representative NPY^+^ and NPY^−^ neurons showing postsynaptic potentials in response to stimulation trains (10 pulses, 40 Hz, stimulus protocol depicted below exemplary traces). NPY^+^ neurons were more likely to generate APs during stimulation trains compared to NPY^−^ neurons (BNST-AL: 5 of 7 NPY^+^ cells from 4 animals versus 1 of 14 NPY^−^ from 4 animals; BNST-AV: 5 of 6 NPY^+^ cells from 3 animals versus 0 of 7 NPY-negative cells from 5 animals). Scale bars in panels **(A–D)**: 5 mV and 25 ms. Stimulation artifacts are clipped in sample traces.

Finally, we investigated synaptic excitability of single neurons in BNST-AL and -AV under current-clamp conditions with intact glutamatergic and GABAergic synaptic transmission. First, we determined the stimulation intensity needed to evoke an AP with afferent, single pulse electrical stimulation. Less stimulation intensity was needed to evoke an AP in NPY^+^ neurons compared with their NPY^−^ counterparts (BNST-AL: 51 ± 9 μA for NPY^+^, *n* = 7/3 versus 134 ± 28 μA in NPY^−^, *n* = 7/3, unpaired *t*-test, *p* < 0.05; BNST-AV: 61 ± 9 μA for NPY^+^, *n* = 7/4 versus 187 ± 33 μA in NPY^−^, *n* = 5/4, unpaired *t*-test, *p* < 0.01, Figures 7A–C). Notably, the stimulation electrode was placed equidistantly when recording from NPY^+^ or NPY^−^ neurons (BNST-AL: 182 ± 14 μm for NPY^+^, and 198 ± 14 μm in NPY^−^, unpaired *t*-test, *p* = 0.49; BNST-AV: 142 ± 19 μm for NPY^+^, and 141 ± 13 μm in NPY^−^, unpaired *t*-test, *p* = 0.97). Second, we tested for AP generation by applying stimulation trains (10 pulses at 40 Hz). NPY^+^ neurons were more likely to generate APs during this stimulation train compared with NPY^−^ neurons (BNST-AL: 5 of 7 NPY^+^ from 4 animals versus 1 of 14 NPY^−^ cells from 4 animals, Fisher’s exact test, *p* < 0.01; BNST-AV: 5 of 6 NPY^+^ cells from 3 animals versus 0 of 7 NPY^−^ cells from 5 animals, Fisher’s exact test, *p* < 0.01, Figure 7D).

## Discussion

The present study shows that NPY^+^ and NPY^−^ neurons in anterior BNST display significant differences in intrinsic electrotonic and electrogenic membrane properties. NPY-expressing neurons possess higher *R*_in_ and lower *C*_in_ and generate APs upon membrane depolarization at an overall higher frequency compared to their NPY-lacking counterparts. Basic properties of a single AP were not different between the two types of cells. Morphologically, NPY^+^ neurons possessed smaller somata, lower total number of processes, lower total dendritic length and less complex branching patterns compared to NPY^−^ neurons in anterior BNST. Furthermore, in comparison to the NPY^−^ subpopulation, NPY^+^ neurons displayed significantly lower GABA_A_ receptor-mediated synaptic responsiveness during evoked, spontaneous, and elementary synaptic activity. A trend toward increased AMPA receptor-mediated responsiveness in NPY^+^ compared to NPY^−^ corroborated the notion of differences in synaptic activity between the two types of neurons. Finally, both NPY^+^ and NPY^−^ neurons in anterior BNST were fitting into the previous classification scheme of type I (Regular Spiking, RS), type II (Low-Threshold Bursting, LTB), and type III (fast Inward Rectifying, fIR) cells (Hammack et al., 2007; Rodríguez-Sierra et al., 2013; Daniel et al., 2017), although the proportion of these physiological phenotypes was similar within the NPY^+^ and NPY^−^ neuronal subpopulation. Finally, with intact glutamatergic and GABAergic synaptic transmission, NPY^+^ neurons displayed higher synaptic excitability compared to NPY^−^ neurons in the anterior BNST.

### Properties of NPY^+^ and NPY^−^ Neurons Related to Neuronal Classification Schemes in BNST

The present study corroborates the findings of Hammack et al. (2007) as well as Rodríguez-Sierra et al. (2013) regarding the dominant classes of cells found in BNST-AL and BNST-AV of the rat. Within both regions of the BNST, the majority of cells (around 2/3) were RS (type I) or LTB (type II) cells. Type II cells accounted for a higher proportion of neurons in BNST-AV than in BNST-AL, matching previous interregional variations in the proportion of cells (Rodríguez-Sierra et al., 2013). Furthermore, the incidence of fIR (type III) neurons has been lower in BNST-AV than in BNST-AL, again consistent with the findings of Rodríguez-Sierra et al. (2013). Further noteworthy is that these classes of neurons were found in different proportions in BNST-AL in different species (Daniel et al., 2017). In the mouse BNST-AL, fIR cells were previously reported to represent the most common cell type accounting for about 54%, while RS cells (type I) accounted for only 15% of the total population (Daniel et al., 2017). An almost inverse proportion has been observed in the present study, with a preponderance of RS cells (around 50%) and only a small proportion (around 20%) of fIR cells in both BNST-AL and BNST-AV. Since both type III and type I cells generate regular series of APs, and scores for *I*_h_ and *I*_K(IR)_ currents obtained under current-clamp conditions overlap in the voltage and time domain, they may segregate into different classes of cells under the various experimental conditions used in different studies. Additional experiments using voltage-clamp recordings combined with pharmacological approaches or expression analyses of transcripts of relevant channel subunits are needed for exact cell classification (Hazra et al., 2011). Of note, the proportion of type I–III cells did not differ across NPY^+^ and NPY^−^ neuronal populations in the anterior BNST. One exception relates to the predominance of LTB type II cells in the population of NPY^−^ negative neurons in BNST-AV. While LTB cells accounted for a higher proportion of neurons also in the rat BNST-AV compared to BNST-AL (Rodríguez-Sierra et al., 2013), low threshold spike bursting characterized NPY-positive interneurons in the mouse striatum (Partridge et al., 2009). Further experimental studies are needed to pinpoint mechanistic correlates of these differences. In any case, NPY^+^ and NPY^−^ neurons comprise the three major classes of neurons (I, II, III) in both BNST-AL and -AV, with relatively few cells remaining unclassified. In characterizing the CRF-tomato transgenic mouse, CRF neurons in the BNST were also described as matching Type I, II, and III cells, although a majority of CRF neurons were not fitting into any of these categories (Silberman and Winder, 2013). Overall, the conclusion is justified that the previous classification scheme and underlying electrophysiological properties are valid for describing electrophysiological phenotypes in anterior BNST, but they are not sufficient for unequivocal identification of NPY or CRF- expressing neurons in these brain regions in mice.

### Physiological Profile of NPY^+^ Neurons in Anterior BNST

While the proportion of type I-III cells was not obviously different between NPY^+^ and NPY^−^ neurons in the anterior BNST, NPY-expressing neurons possessed higher *R*_in_ and lower *C*_in_, and generated series of spikes upon depolarization at higher frequencies compared to their NPY-lacking counterparts. Similar observations were made in the PFC, where all NPY-GFP neurons were fast spiking with only mild frequency adaptation (Saffari et al., 2016). The NPY^+^ GABAergic neurons in PFC could be categorized into three main morphological classes, with short multipolar cells being by far the most frequently encountered form (Saffari et al., 2016). Similar observations were made in striatum, where NPY^+^ GABAergic interneurons were found to have fusiform cell bodies bearing sparsely ramified dendrites and to generate spontaneous series of APs at high frequencies eventually leading to burst firing (Partridge et al., 2009). Fast spike firing rates and cell bodies bearing short dendritic processes thus seem to be features of NPY^+^ neurons in various regions of the mouse brain. Accordingly, in this study NPY^+^ neurons in the anterior BNST displayed high-frequent spike activity, and smaller somata giving rise to shorter, less ramified and less complex dendritic arbors compared to their NPY^−^ counterparts. In line with previous reports, the majority of neurons in the anterior BNST bore dendritic varicosities (Larriva-Sahd, 2006; Rodríguez-Sierra et al., 2013; Gungor et al., 2018). It is noteworthy that the ratio of membrane surface area across the various compartments of a neuron can influence its firing properties, even without a change in types and densities of voltage-gated ion channels (Mainen and Sejnowski, 1996). With all properties unchanged, a decrease in the ratio of dendritic to axon-somatic surface membrane area results in an increase of spike firing rates. A limited dendritic membrane surface area may thus contribute to the high spike firing rate in NPY^+^ neurons. In more general terms, differences in membrane surface area of the dendritic and somatic neuronal compartments may help to explain the typifying tonic series of spike firing in NPY^+^ and NPY^−^ neurons observed in anterior BNST. The expression pattern of voltage-gated ion channels and their subtypes, mediating *I*_T_, *I*_h_, and *I*_K(IR)_ currents (Hazra et al., 2011), may then determine the electrophysiological phenotype of the various subclasses across both NPY^+^ and NPY^−^ neuronal populations.

A limited dendritic membrane surface area may also contribute to the relatively low synaptic responsiveness observed in NPY^+^ neurons as compared to NPY^−^ neurons. The differences in amplitude of both sIPSCs and mIPSCs that were found between the two types of neurons suggest involvement of postsynaptic sites, with low amplitude values in NPY^+^ neurons likely reflecting a limitation in number and/or recruitment of postsynaptic GABA_A_ receptors. In keeping with this, eIPSCs were of low amplitude in NPY^+^ neurons as compared to those in NPY^−^ neurons. While the GABAergic input pathways activated upon local electrical microstimulation in the present study remain unidentified, two lines of findings are worth mentioning. First, GABAergic neurons are the prevalent type of neurons in both BNST-AL and BNST-AV (Cullinan et al., 1993), with the latter containing a low proportion of glutamatergic cells (Poulin et al., 2009), some of which are projection neurons (Kudo et al., 2012). GABAergic neurons in BNST-AL and BNST-AV are mutually interconnected (Turesson et al., 2013; Gungor and Paré, 2016), and BNST-AL and BNST-AV receive strong GABAergic innervation from CeL and, to a minor extent, from CeM (Sun and Cassell, 1993; Li et al., 2012; Wood et al., 2015; Gungor and Paré, 2016). In BLA, more than 80% of NPY^+^ neurons also express GABA, and virtually all NPY cells co-express SST (McDonald, 1989). Most of the NPY neurons in CeL had initially been found to not co-localize with SST and had been considered local GABAergic neurons (Gustafson et al., 1986; McDonald, 1989). A more recent study using NPY-GFP mice has demonstrated that NPY in the CeA is contained in SST neurons, in particular in CeM, and that a portion of these neurons project to BNST (Wood et al., 2015). Overall, the conclusion seems justified that NPY^+^ neurons in BNST-AL and -AV are GABAergic, receive GABAergic inputs from both local BNST neurons and GABAergic projections from CeA, and that GABAergic responsiveness is relatively low as compared to NPY^−^ neurons in the same areas. Second, BNST receives few exteroceptive sensory afferents via the thalamus and cortex, but gets massive glutamatergic projections from the basolateral complex, with the basal nucleus heavily projecting to BNST-AL and -AV (as reviewed by Gungor and Paré, 2016). Differences observed in glutamatergic transmission between NPY^+^ and NPY^−^ neurons were much less prominent compared to those in GABAergic synaptic transmission. The only difference reaching statistical significance was between probabilities of mEPSCs. Therefore, it is interesting to conclude that GABAergic NPY-expressing neurons in BNST-AL and -AV, compared to NPY-lacking neurons, possess a relatively low responsiveness to GABAergic inputs, including those from local BNST neurons and CeA afferents, whereas glutamatergic responsiveness differs much less between the two populations of neurons. These properties, together with the intrinsic capability of firing high frequent series of APs upon membrane depolarization, may constitute an overall high state of excitability in NPY^+^ neurons in anterior BNST.

### Possible Functional Impact of NPY^+^ Neurons in Anterior BNST

A vast literature supports the view that NPY is a major neurochemical component of the stress response, coordinating neuronal, vascular, immune, and metabolic functions (Heilig, 2004; Rasmusson et al., 2010; Tasan et al., 2016). Overall, the NPY system is considered to adapt the organism to stressful, potentially life-threatening conditions and to maintain physiological integrity, in both rodents (Cohen et al., 2012) and humans (Wu et al., 2013). While relatively few studies have investigated the effects of NPY in the BNST, there is evidence indicating that NPY can modulate inhibitory GABAergic input or directly hyperpolarize BNST neurons depending on whether pre- or postsynaptic receptors are stimulated (Tasan et al., 2016). For instance, stimulation of presynaptic Y2 receptors reduces GABAergic transmission to BNST-AV neurons (Kash and Winder, 2006; McCall et al., 2013). Chronic stress impairs this ability of NPY to suppress IPSCs in DBA/2J mice, but not in C57BL/6J mice, suggesting that stress can alter NPY signaling in BNST depending on genetic background (Pleil et al., 2012). Stimulation of postsynaptic NPY receptors of the Y1 or Y5 subtype induces a negative shift in RMP in a subset of BNST-AL neurons by blocking the *I*_h_ current (Ide et al., 2013).

On the systems level, the BNST, as part of the extended amygdala, is considered a center of valence monitoring by integrating information with negative valence or anxiety-like states, and has recently gained attention as a relevant region for human stress-related psychiatric diseases (Walker et al., 2009; Lebow and Chen, 2016). Much focus has been on the anterior BNST, given that it is the main termination zone of axonal inputs from central and basal amygdala (Gungor and Paré, 2016), and these connections reportedly play a crucial role in mediating behavioral processes related to fear and anxiety (Fendt et al., 2003; Walker et al., 2003; Tye et al., 2011; Tovote et al., 2015). Early studies had already suggested the existence of two related but dissociable fear response systems with the CeA mediating rapid stimulus-specific responses to imminent threats and the anterior BNST generating lasting responses to more ambiguous threats (Walker et al., 2003). This simplifying view has been replaced based on more recent findings from both animal and human studies (see Gungor and Paré, 2016; Fox and Shackman, 2017), showing that the BNST is involved in organizing fear responses to stress-related stimuli that are poorly predictable in terms of onset, duration or complexity (reviewed by Goode and Maren, 2017). Optogenetics combined with loss-of-function approaches have indeed identified distinct axonal pathways from amygdala to anterior BNST, which mediate anxiety-like responses to environmental stimuli bearing temporally unpredictable threat (Lange et al., 2016). It is noteworthy that synaptic activity at these distinct connections in anterior BNST is down-regulated in an activity-dependent manner through stimulation of presynaptic cannabinoid CB1 receptors, which in turn is causal for generation of anxiety-like behavior (Lange et al., 2016). The relevant CB1-regulated projections originate from basal and centrolateral amygdalar nuclei and connect to BNST-AL and BNST-AV, while largely sparing BNST-AM and BNSTov. It is interesting to note that these target areas largely coincide with the sites of high levels of NPY^+^ neurons in anterior BNST.

This, together with the accepted view that the NPY system adapts the organism to stressful and potentially life-threatening conditions (as discussed above), suggests that NPY^+^ neurons in anterior BNST may be activated upon stress-related stimuli bearing unpredictable contingencies. Release of NPY will then dampen activity in BNST neurons through both pre- and postsynaptic mechanisms, mediated via Y2 and Y1 receptors, respectively (as discussed above), which will limit anxiety-like responses. Two lines of evidence support this view. First, there is a strong link between CRF and anxiety in BNST (for review see Daniel and Rainnie, 2016). The anxiogenic influence of CRF largely (but not exclusively, see Kash and Winder, 2006; Ide et al., 2013) involves CRF1 receptor-mediated potentiation of glutamatergic transmission to BNST-AL (Kash et al., 2008; Nobis et al., 2011; Silberman et al., 2013), likely including CeL inputs (Sakanaka et al., 1986; Jaferi and Pickel, 2009; Jaferi et al., 2009). Of note, optogenetic silencing of a CRF pathway from CeL to BNST-AL disrupted sustained-type of fear responses in a contextual training paradigm (Asok et al., 2017), extending earlier findings on a critical role of the CRF system in amygdala-BNST pathways related to unpredictable threat and sustained fear responses (Davis et al., 2010; Daniel and Rainnie, 2016). Second, NPY and CRF have largely opposing effects on BNST neuronal activity and behavioral impact. An early slice study in BNST neurons showed that Y2 receptor stimulation suppresses, while CRF1 receptor stimulation enhances GABAergic synaptic transmission (Kash and Winder, 2006). Furthermore, injection into the dorsolateral BNST of a CRF1 or CRF2 receptor antagonist suppressed aversive responses in a conditioned place aversion test, while NPY injection suppressed aversion mediated via Y1 or Y5 receptors (Ide et al., 2013). The effects of CRF and NPY were associated with increased and decreased neuronal excitability in type II neurons in BNST-AL (Ide et al., 2013). Interestingly, the NPY and CRF system seems to exert opposing functions in the regulation of various emotional and reward-seeking behaviors (see for example Pleil et al., 2015). In any case, future studies should characterize the specific neuronal and mechanistic substrates of NPY-CRF interactions in anterior BNST, and their relevance for adaptation to stressful encounters in an unpredictable environmental context.

## Conclusion

In conclusion, these properties indicate an overall state of high excitability in NPY^+^ neurons in the anterior BNST. Given the crucial role of both the anterior BNST and the NPY system in fear and defensive responses to threat stimuli, these findings suggest a scenario where NPY^+^ neurons in BNST are preferentially active and responsive to afferent inputs, and thereby contribute to adaptation of the organism to stressful environmental encounters.

## Author Contributions

H-CP, MDL, ALW, and JCB were responsible for conception of the study and design of the experiments. ALW, MD, JCB, and MDL acquired and analyzed the data. ALW, H-CP, JCB, PB, and MDL were involved in interpretation of the data and drafting it for the manuscript. All authors contributed to the final editing of the manuscript, approved the final version and therefore agree to be accountable for all aspects of the work.

## Conflict of Interest Statement

The authors declare that the research was conducted in the absence of any commercial or financial relationships that could be construed as a potential conflict of interest.

## Abbreviations

AC: anterior commissure
ACSF: artificial cerebrospinal fluid
AMPA: α-amino-3-hydroxy-5-methyl-4-isoxazolepropionic acid
AP: action potential
BAC: bacterial artificial chromosome
BLA: basolateral amygdala
BNST: bed nucleus of stria terminalis
BNST-AL: anterolateral BNST
BNST-AM: anteromedial BNST
BNST-AV: anteroventral BNST
BNSTov: oval nucleus of the BNST
CeA: central amygdala
CeL: centrolateral amygdala
CeM: centromedial amygdala
C_in_: membrane capacitance
CRF: corticotropin releasing factor
dNTP: deoxyribonucleotide triphosphate
(s/m/e) EPSC: (spontaneous/miniature/evoked) excitatory postsynaptic current
fIR: fast Inward Rectifying neurons (type III)
GABA: γ-aminobutyric acid
GFP: green fluorescent protein
HPRT: hypoxanthine-phosphoribosyltransferase
(s/m/e) IPSC: (spontaneous/miniature/evoked) inhibitory postsynaptic current
ISI: inter-stimulus interval
K-gluconate: potassium gluconate
LTB: Low-Threshold Bursting neurons (type II)
MP: membrane potential
NPY: neuropeptide Y
PBS: phosphate-buffered saline
PCR: polymerase chain reaction
PFA: paraformaldehyde
PFC: prefrontal cortex
PP: pancreatic polypeptide
PVN: hypothalamic paraventricular nucleus
PYY: peptide YY
*R*_in_: input resistance
RM: repeated measures
RMP: resting membrane potential
RMS: root mean square
RS: Regular Spiking neurons (type I)
*R*_s_: series resistance
RT: reverse transcriptase reaction
SST: somatostatin
TTX: tetrodotoxin.
